# Pervasive subduction zone devolatilization recycles CO_2_ into the forearc

**DOI:** 10.1038/s41467-020-19993-2

**Published:** 2020-12-04

**Authors:** E. M. Stewart, Jay J. Ague

**Affiliations:** 1 Department of Earth and Planetary Sciences, Yale University, 210 Whitney Avenue, New Haven, CT 06511 USA; 2 Present Address: California Institute of Technology, Division of Geological and Planetary Sciences, 1200 E California Boulevard, Pasadena, CA 91125 USA

**Keywords:** Carbon cycle, Geochemistry, Petrology

## Abstract

The fate of subducted CO_2_ remains the subject of widespread disagreement, with different models predicting either wholesale (up to 99%) decarbonation of the subducting slab or extremely limited carbon loss and, consequently, massive deep subduction of CO_2_. The fluid history of subducted rocks lies at the heart of this debate: rocks that experience significant infiltration by a water-bearing fluid may release orders of magnitude more CO_2_ than rocks that are metamorphosed in a closed chemical system. Numerical models make a wide range of predictions regarding water mobility, and further progress has been limited by a lack of direct observations. Here we present a comprehensive field-based study of decarbonation efficiency in a subducting slab (Cyclades, Greece), and show that ~40% to ~65% of the CO_2_ in subducting crust is released via metamorphic decarbonation reactions at forearc depths. This result precludes extensive deep subduction of most CO_2_ and suggests that the mantle has become more depleted in carbon over geologic time.

## Abstract (web summary)

The fate of subducted CO_2_ remains debated, with estimates mainly from numerical predictions varying from wholesale decarbonation of the shallow subducting slab to massive deep subduction of CO_2_. Here, the authors present field-based data and show that ~40% to ~65% of the CO_2_ in subducting crust is released via metamorphic decarbonation reactions at forearc depths.

## Introduction

Subduction of oceanic lithosphere is arguably the most important process in global geochemical cycling. Subduction delivers material from Earth’s surface into the deep mantle, but chemical reactions in the subducting slab can also release volatiles which ultimately return to the atmosphere^1–3^. There is, however, a long-standing debate surrounding the fate of subducted CO_2_. Recent papers have argued that the vast majority of oceanic carbon is deeply subducted^4^ or that the vast majority is released by subarc depths^2^.

Carbon dioxide is released during metamorphism when a carbonate mineral such as aragonite or dolomite breaks down via prograde decarbonation reactions^5^. A model reaction is 2 Quartz + 3 Dolomite + 2 Albite + H_2_O = 3 Aragonite + Glaucophane + 3 CO_2_. For decades, thermodynamic models of subducted rocks have been used to estimate the degree of carbon loss^6–10^. However, the modeling results vary widely, due mostly to uncertainties on the nature of aqueous fluid infiltration into subducting rocks. Closed-system models predict evolution under relatively water-poor conditions^6,7^, whereas models that consider open-system fluid infiltration allow for more water-rich environments^8–10^. The presence of a water-bearing fluid has a profound effect on carbonate stability, decreasing reaction temperatures by as much as hundreds of Kelvin^11^. Thus, it is essential to constrain the nature and extent of fluid infiltration to estimate the resultant CO_2_ release.

Given a lack of observational constraints, models predict that anywhere from ~0.0001 to 52 Megatons of oceanic carbon will be deeply subducted annually^2^. These large uncertainties in our current understanding of CO_2_ subduction leave basic questions about global geochemical cycling unanswered. For example, has the deep Earth become depleted or enriched in carbon over geologic time? To what extent might enhanced carbonate subduction add CO_2_ to Earth’s atmosphere? Providing answers to such questions requires a new constraint on the fate of subducted CO_2_.

Here, we provide an extensive field-based record of decarbonation efficiency in a subducted slab. We combine detailed observations, stable isotope measurements, and bulk chemical analysis with thermodynamic modeling to show that subducted rocks from the Cycladic Blueschist Unit in Greece underwent major fluid infiltration and decarbonation. Our results show that at least ~40%, and more likely ~65%, of the CO_2_ in the typical subducting oceanic crust is released at forearc depths by decarbonation reactions. Other processes, such as stoichiometric carbonate dissolution^4^, would add to the total. Whether the released carbon is ultimately stored in the overriding lithosphere or delivered to Earth’s atmosphere, much of it is not returned to the deep mantle, which, as a consequence, has likely become more depleted in carbon over geologic time.

## Results

### Geologic background

The Cycladic Blueschist Unit (CBU) in Greece is an ideal field area to test existing models of subduction zone decarbonation. It was metamorphosed in the Eocene when the African plate subducted northward beneath Eurasia^12–14^. Rocks reached peak temperatures (*T*) of ~500 to ~550 °C and peak pressures (*P*) of ~1.5 to 2.0 GPa^15–18^. Preservation of subduction-related features in the CBU is superlative—Syros Island in particular is home to one of the best-preserved and most thoroughly studied subduction complexes on Earth. Apart from its exceptional preservation, the CBU represents a normal subduction complex, with multiple studies using observations of the CBU for inferences about subduction in general^19–23^.

We collected more than 600 hand samples of carbonate-bearing rocks from the islands of Syros and Tinos (see “Methods”; Supplementary Fig. 1). These can be broadly categorized in four groups according to their protolith: carbonate-bearing siliciclastic rocks, limestones, heavily altered basaltic volcanics, and more lightly altered basaltic oceanic crust. Of these, 211 were selected for carbon and oxygen isotope analysis. A subset of 56 representative rocks underwent detailed geochemical analysis including major, minor, and trace element chemistry (Supplementary Data 1) and thermodynamic modeling (see “Methods”; Supplementary Table 1). These rocks were selected as the best-preserved representatives of the breadth of lithologies in the CBU, with no evidence of appreciable metamorphic retrogression during exhumation.

### Estimating CO_2_ loss

Samples preserve their mineralogy, and therefore mineral-bound CO_2_ content, from peak *P–T* conditions. In order to estimate the fraction of carbon released by each sample (∆CO_2_), we compare its modern CO_2_ content to its composition before major metamorphic devolatilization began. The modern CO_2_ content is easily measured (see “Methods”), but the initial volatile content is not directly preserved. Thus we need a technique to determine how much CO_2_ was in each rock prior to metamorphic decarbonation.

A new modeling approach is used to backproject this initial volatile content for each sample. We calculate the phase equilibria for each rock with a CO_2_-bearing fluid present in excess at lower grade *P–T* conditions (310 °C and 0.8–1.0 GPa) consistent with estimates of the prograde *P–T* path on Syros^16^ and warm subduction in general^24,25^. The initial back-projected composition is then taken as the composition of the solid minerals at these lower *P–T* conditions. In most samples, the modeled CO_2_ content is insensitive to changes in P, T, or fluid mole fraction of CO_2_ ($X_{{\mathrm{CO}}_{2}}$) in this lower *P–T* range. For ten samples that do show significant variation, we select the minimum plausible initial CO_2_ content, ensuring that our estimated results for ∆CO_2_ are also minima and, thus, are conservative (see “Methods”; Supplementary Fig. 3).

The back-projection method requires only three assumptions. (1) Rocks were in equilibrium with a CO_2_-bearing fluid (regardless of its $X_{{\mathrm{CO}}_{2}}$) at lower *P–T* conditions. (2) Rocks that contained carbonate at peak *P–T* contained an equal or greater mass of carbonate prior to metamorphism. (3) Rocks did not undergo major element metasomatism other than volatile loss. The most likely metasomatic process is stoichiometric carbonate dissolution^26^ which would add to the total CO_2_ released. Thus, once again, our estimates for ∆CO_2_ are conservative.

### Forward modeling metamorphism

With this initial composition, forward modeling is performed for two end-member scenarios: (1) a fully closed system^6,7^ and (2) a system open to fluid infiltration, approximated with a fixed-$X_{{\mathrm{CO}}_{2}}$ fluid in excess^27^ (see “Methods”). Fully closed-system models are run at peak *P–T* conditions (525 °C and 1.5 GPa) to estimate the resulting rock and fluid compositions. Fluid-infiltrated models use observations of the mineral assemblage in each sample (see “Methods”; Supplementary Table 1) in combination with calculated pressure–activity of CO_2_ diagrams to constrain the equilibrium fluid composition ($X_{{\mathrm{CO}}_{2}}$) recorded by each sample at peak *P–T*.

Furthermore, we estimate the maximum plausible CO_2_ loss for each sample. The mineral assemblage is calculated at peak conditions with a very water-rich ($X_{{\mathrm{CO}}_{2}}$ = 0.0001) fluid in excess. The CO_2_ content of this assemblage is then compared to the back-projected wt% CO_2_ to calculate the potential (or maximum) ∆CO_2_ of each sample.

Using higher *P–T* values of 550 °C and 2.0 GPa does not impact our conclusions (see below).

### Evidence for fluid infiltration

**Fig. 1:**
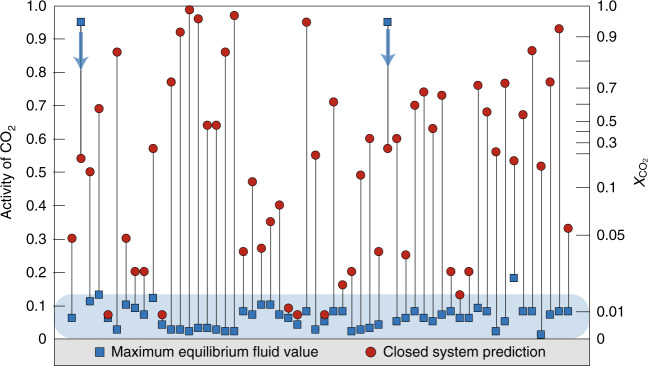
Equilibrium fluid compositions. Blue squares show maximum possible fluid CO_2_ activity of each sample. The equivalent $X_{{\mathrm{CO}}_{2}}$ is shown on the right axis. Note the nonlinear relationship between activity of CO_2_ and $X_{{\mathrm{CO}}_{2}}$. Red circles are CO_2_ activities achieved in a closed system. The vast majority of samples record water-rich fluids, inconsistent with evolution in a closed system. Two samples are unconstrained, with any CO_2_ activity < ~0.95 being plausible (blue arrows). The shaded area shows the 2σ standard deviation range of equilibrium activity values (0.06 ± 0.07), excluding the two unconstrained outliers.

The equilibrium fluid compositions required by rock mineralogy are consistently water-rich. Figure 1 shows the maximum activity of CO_2_ ($a_{{\mathrm{CO}}_{2}}$) recorded by each sample. In nearly all cases, the mineralogy requires low-CO_2_ (i.e., water-rich) conditions ($a_{{\mathrm{CO}}_{2}}$ = 0.06 ± 0.07 2σ standard deviation). Because CO_2_–H_2_O mixing is highly nonideal at subduction zone conditions, this corresponds to an even lower $X_{{\mathrm{CO}}_{2}}$, averaging ~0.006. Two outliers are excluded from the above statistics because they contain very simple mineral assemblages that offer almost no constraints on the equilibrium fluid compositions (0 < $a_{{\mathrm{CO}}_{2}}$ < 0.95).

In contrast, closed-system modeling of these samples systematically predicts fluid compositions that are clearly richer in CO_2_ (Fig. 1). This demonstrates that the water-rich conditions recorded by most of the rocks could not be achieved through evolution in a closed system, and therefore required open-system infiltration of a water-bearing fluid.

**Fig. 2:**
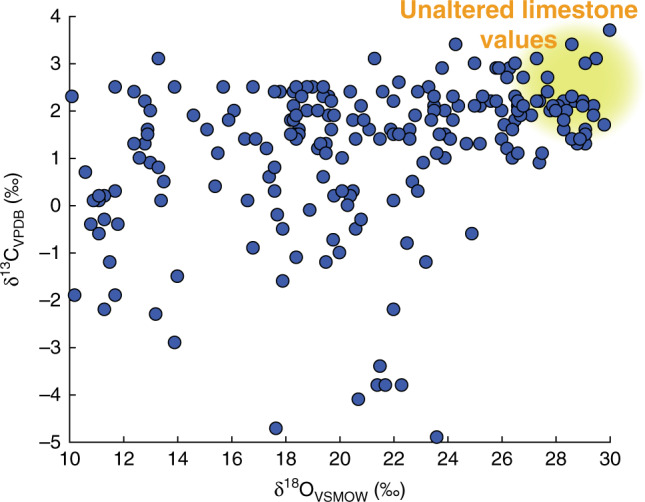
Carbon and oxygen isotopes. The 211 isotope measurements of metamorphic carbonate minerals record a wide array of δ^13^C and δ^18^O values that differ from unaltered seafloor carbonate. Uncertainties of <0.1‰ (1σ) are smaller than plotted circles.

Carbon and oxygen isotope data show a wide range of values from δ^13^C_VPDB_ = −4.9 to +3.7 ‰ and δ^18^O_VSMOW_ = +10.1 to +30.8 ‰ (Fig. 2). While many of these samples lie within the range expected for seafloor carbonates (δ^13^C_VPDB_ = ~+1 to +4 ‰ and δ^18^O_VSMOW_ = ~+26 to +31 ‰)^28^, most (>70%) record significantly lighter C and O isotope signatures.

Some of the variations in δ^18^O may result from the prograde exchange with local silicates^29^, but very low δ^18^O must be attributed to external fluid infiltration^26^, perhaps up-temperature fluid flow in particular^30^. Low δ^13^C values may indicate the presence of reduced carbon, isotope fractionation as a result of high degrees of decarbonation^31^, or infiltration by a low δ^13^C fluid.

Dewatering of subducted serpentinite or other hydrated lithologies (e.g., metasediments) is expected to release substantial water^9,32,33^; we conclude that this is the most likely source of the water-bearing fluid which eventually traveled into the carbonate rocks, perhaps as part of a channelized flow system^34^, altering isotopic compositions and phase equilibria.

### Decarbonation efficiency

**Fig. 3:**
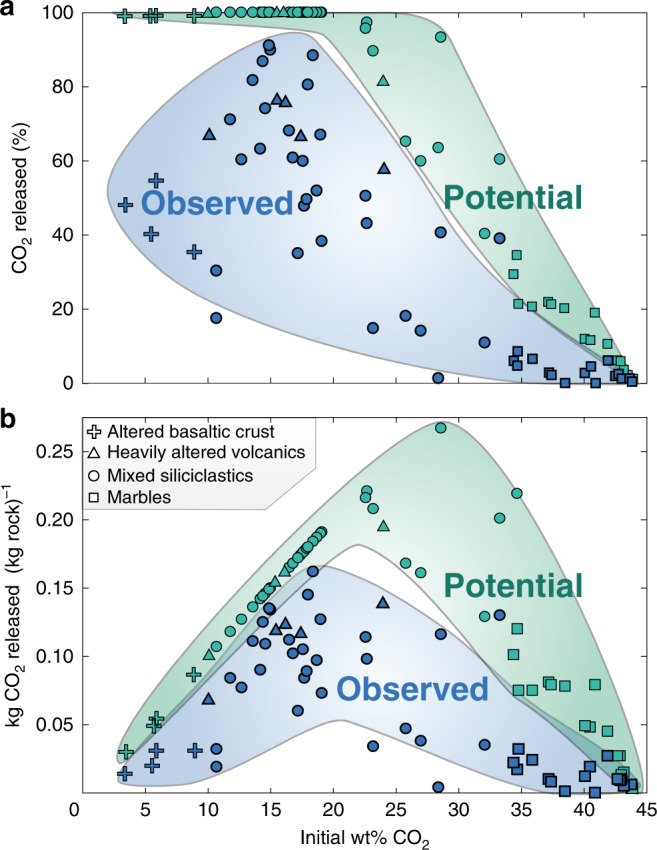
Degree of decarbonation. Blue symbols show the observed CO_2_ loss of each sample whereas green symbols show the maximum potential CO_2_ loss as a function of initial wt% CO_2_. **a** Percent of CO_2_ released. Nearly pure carbonate rocks degas the smallest proportion of CO_2._ **b** The kg CO_2_ released per kg rock. This shows a peaked distribution, with intermediate-CO_2_-content rocks degassing the greatest mass of CO_2_ in both observed and potential datasets.

Rocks record a wide array of observed decarbonation (∆CO_2_) values, ranging from 0 to 91% CO_2_ loss by mass (Fig. 3a). Importantly, these values do not depend on the $X_{{\mathrm{CO}}_{2}}$ estimates. The data define a consistent trend: very pure marbles starting with ~40 wt% CO_2_ released little to no CO_2_ while more carbonate-poor rocks, particularly mixed carbonate-siliciclastic metasediments, underwent extensive decarbonation. The potential decarbonation values for the same samples range from 0 to 100% CO_2_ loss. In terms of the mass of CO_2_ produced, both the observed and potential datasets show a peaked distribution (Fig. 3b), with intermediate-composition rocks (whether metasedimentary or metavolcanic) releasing the greatest mass of CO_2_. Conversely, nearly pure limestone and relatively carbonate-poor altered oceanic crust release an appreciably smaller mass of CO_2_.

The results shown in Fig. 3 comprise a comprehensive field-based record of decarbonation efficiency in subducted lithologies. One implication for global carbon cycling is that the degree of decarbonation varies markedly from one rock type to the next. Thus, two subduction zones that subduct an identical mass of CO_2_ per year could have dramatically different CO_2_ fluxes off the slab, depending on whether CO_2_ is hosted in, for instance, pure limestones or heavily altered volcanics. For this reason, phase equilibria modeling of decarbonation of a composite or average rock composition^6–8^ is unlikely to illuminate the full spectrum of devolatilization behavior during subduction. It is essential to know the local chemical environment of subducted carbon to predict whether it is devolatilized or retained within the slab.

### Global carbon mobility

Using this record of major, but variable, metamorphic decarbonation, we can consider the efficiency of carbonate subduction in individual modern or ancient subduction zones or the Earth system as a whole. For example, we calculate the proportion of CO_2_ released from a fictive slab that is roughly equivalent to the global average subducting crust, with 25 m of carbonate-bearing siliciclastics, 25 m of limestone, 50 m of heavily altered volcanics, and 200 m of lightly altered oceanic crust. This gives a global input of CO_2_ into subduction zones of 5.08 Tmol CO_2_ yr^−1^, well within the range of lower and upper bounds from refs. ^2,3^.

The global average flux from subducting slabs, *F*, can then be expressed according to Eq. (1):

1 $$F = {\int}_{z_1}^{z_2} {C_{{\mathrm{CO}}_2} \cdot L \cdot R \cdot \rho \cdot {\Delta}{\mathrm{CO}}_2^{{\mathrm{observed}}} \cdot dz},$$

where *z* is depth, $C_{{\mathrm{CO}}_{2}}$ is the initial concentration of CO_2_, *L* is the global trench length, *R* is the global average convergence rate, and *ρ* is rock density. For this global average crust, we find *F* = 2.06 Tmol CO_2_ yr^−1^, corresponding to ~40% CO_2_ loss. As the rocks of the CBU record maximum depths of ~50 to ~70 km, this flux of CO_2_ is generated in the forearc region.

### Pulsed decarbonation

**Fig. 4:**
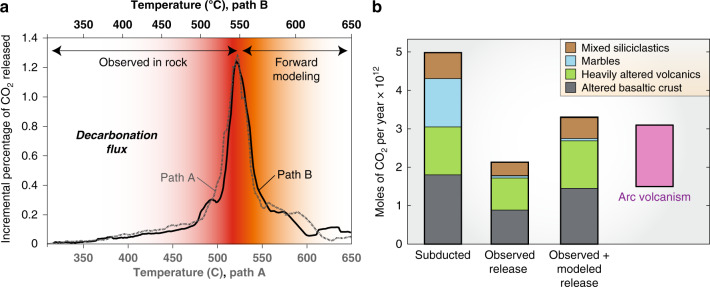
Carbon flux. **a** The percentage of CO_2_ degassed along two linear *P–T* paths. Both paths begin at 310 °C and 1.0 GPa; path A continues through peak field conditions of 525 °C and 1.5 GPa, while path B passes through 550 °C and 2.0 GPa. Paths A and B end at 650 °C and 1.8 or 2.4 GPa, respectively. A sharp pulse of decarbonation is seen around peak-*T* conditions. **b** The constructed moles of CO_2_ subducting globally and the amount of CO_2_ released at both peak conditions (observed) and up to 650 °C (observed + modeled). The arc volcanic CO_2_ flux^1^ is shown for reference.

We also consider where along the subduction path this CO_2_ was devolatilized. We forward model CO_2_ loss in three representative samples for each of the four general lithologies for a total of 12 detailed forward models. Models are calculated along two linear *P–T* paths spanning the range of possible metamorphic conditions on Syros and Tinos. The modeling differs considerably from previous approaches in that it is constrained by the fluid compositions and decarbonation extent recorded by natural subducted samples (see “Methods”). This allows us to calculate (1) the *P–T* conditions at which decarbonation occurred, (2) the rate of CO_2_ release, and (3) how decarbonation may have proceeded if the rocks had continued to subduct. Using the same global average crustal stack, we generate curves showing the percentage of the initial CO_2_ released at each *P–T* step with a 20 °C moving mean (Fig. 4).

A sharp pulse of decarbonation is seen near the observed maximum *P–T* conditions (Fig. 4a), with relatively little carbon loss before or after this pulse. This is true for both the hotter and colder *P–T* paths, with the colder model predicting CO_2_ release of approximately the same magnitude, just at greater depth. Thus our results suggest that, for rocks to undergo the ~40% decarbonation which we observe, most of the CO_2_ would need to be devolatilized in a pulse around ~500–550 °C.

This is new field-based evidence for pulsed decarbonation, which had previously been predicted with a purely modeling-driven approach^8^. Furthermore, by extending these models beyond the observed *P–T* conditions, we predict that the rocks would have lost an additional ~25 to 30% of their initial CO_2_ had they continued to subduct to 650 °C and 1.8 to 2.4 GPa, conditions still representing forearc depths.

There is an intriguing coincidence between the location of this pulse and peak *P–T* conditions. This is not an artifact of our model but rather suggests that there may be some link between devolatilization, exhumation processes, and/or kinetic preservation of the observed mineralogy. Further work is needed to determine whether a similar coincidence can be observed in other exhumed slabs.

### Heterogeneous subduction of carbon

Because the proportion of CO_2_ devolatilized is heavily dependent on the subducting lithologies, we expect the degree of decarbonation to vary markedly from one subduction zone to another. As an example, we calculate the CO_2_ loss for two end-member modern subduction zones: Central America and Tonga. The subducting material beneath Central America is dominated by relatively pure CaCO_3_ limestones atop young (~23 Ma) altered oceanic crust. Assuming a crustal stack with 250 m of limestone (a rough approximation of the sediments recorded at DSDP site 495)^35^, and 100 m of lightly altered oceanic crust, we predict only ~6% CO_2_ loss by 550 °C, much less than the global average of ~40%. Tonga represents the other extreme, with essentially no carbonate-bearing sediments, and nearly all subducted CO_2_ hosted in lightly altered oceanic crust^36^. We would, therefore, predict that metamorphic reactions would release ~45% of the rock’s CO_2_ at comparable conditions. The differences become even more pronounced in the projection to 650 °C, where Central American and Tongan subducted lithologies are predicted to have undergone ~10% and ~95% decarbonation, respectively. These results are fully consistent with those predicted on a conceptual basis by ref. ^3^.

## Discussion

Rocks of the CBU record a robust and consistent record of substantial aqueous fluid infiltration which drove metamorphic decarbonation. The CO_2_ loss ranges considerably, but systematically, across rocks of different compositions. Assuming lithologic proportions representing average oceanic crust and sediments, these rocks record that ~40% of subducted CO_2_ was released. Forward modeling predicts an additional ~25% CO_2_ loss into the forearc, with most decarbonation concentrated in a sharp peak.

**Fig. 5:**
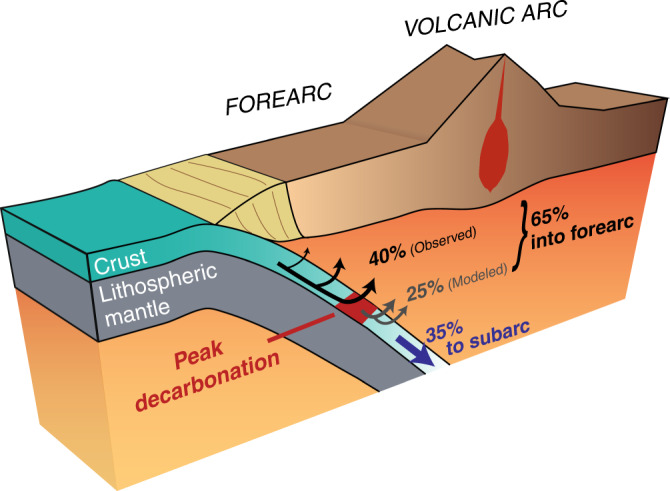
Global mass balance. This schematic representation shows the ~40% CO_2_ loss recorded in the field and the total predicted 65% CO_2_ loss by 650 °C in the forearc; most decarbonation is concentrated in a sharp peak at ~500–550 °C. The remaining ~35% of subducted CO_2_ in the slab continues to greater depths where it could be released by processes such as dissolution or melting, or retained in the slab past the subarc. Global estimates are based on observations from the Cycladic Blueschist Unit.

This leaves ~35% of the initial CO_2_ in the slab to travel to subarc depths and, perhaps, beyond (Fig. 5). As noted above, this global average is not necessarily representative of any specific subduction zone, with subduction beneath Central America or Tonga expected to deliver 90% or 5% of crustal CO_2_ to the subarc, respectively.

Other processes such as melting^37^ or diapirsm^38^ could add to the amount of CO_2_ released. In particular, our approach does not quantify carbonate dissolution^26,39^, which was likely only beginning at the depths reached by the CBU, and could have been much more extensive at greater depths^26^. Slab dehydration continues well into the subarc^40^, providing the fluids needed to drive dissolution and melting processes.

It is important to consider how well the CBU represents subduction in general. Indeed, it has been suggested that the entire global suite of exhumed subduction complexes may be anomalously warm relative to average subduction^25,41^. The CBU is, in this sense, certainly not representative of thermal conditions in all subduction zones. Nevertheless, we find that a similar degree of decarbonation is achieved along differing *P–T* paths (Fig. 4); CO_2_ release is simply delayed to greater depths along a colder geotherm. Thus we expect that colder or hotter subduction zone paths will result in similar total degrees of decarbonation, provided only that a water-bearing fluid is present.

Once released, the fate of devolatilized CO_2_ is uncertain. A substantial proportion may be stored in the lithospheric mantle of the overriding plate; the mass of CO_2_ release we calculate is greater than most estimates of the CO_2_ flux from arc systems into Earth’s atmosphere. This is consistent with recent evidence for major carbonate precipitation in forearc lithosphere through both surface measurements^42^ and observation of carbonated ultramafic lithologies from the slab or mantle wedge^43,44^. In fact, a deep lithospheric CO_2_ reservoir may be required to reconcile our results with global mass-balance arguments^4^. On the other hand, some CO_2_ will escape to Earth’s atmosphere as part of a diffuse CO_2_ flux and/or by contributing to arc volcanic CO_2_ emissions^2^.

The strong compositional dependence of decarbonation suggests the proportion of CO_2_ release will vary across space and time depending upon what rocks are being subducted. While the thermal structure of a given subduction zone will control the depth at which these decarbonation reactions occur, we predict that the same total degree of CO_2_ loss may be expected over a wide range of *P–T* paths—the caveat being that fluid infiltration is essential to drive decarbonation, thus variability in fluid mobility across different subduction regimes may result in very different degrees of decarbonation.

We predict a relatively small proportion of CO_2_—a maximum of ~35% globally—will be retained in the slab past forearc depths. Thus, subduction is inefficient at returning CO_2_ to the mantle: our model predicts less than ~2 Tmol CO_2_ yr^−1^ is subducted past the forearc. This is comparable to the amount released by arc volcanism annually^1^, but whether it is the source of arc CO_2_ is an outstanding open question. Decarbonation reactions would be unable to release this CO_2_ in the subarc (Fig. 4), which means that other processes, such as sediment melting, congruent dissolution, or diapirism, would have to operate there. In addition, mantle corner flow could, perhaps, transport carbonated forearc mantle into the subarc melting region^8^. Consequently, we do not consider evidence of slab-derived CO_2_ in volcanic arcs^45^, to be at odds with our observations.

Regardless of whether the ~2 Tmol CO_2_ yr^−1^ is released by arcs or subducted deeper into the mantle, it is far less than recent estimates of mantle-derived CO_2_ outgassing, totaling ~5 to ~7 Tmol CO_2_ yr^−1^ ^3^. Therefore, CO_2_ fluxes into and out of Earth’s convecting mantle appear out of balance on the modern Earth. If this imbalance has persisted on long timescales, we posit that the deep mantle has been progressively depleted in carbon through Earth history, and it may lose more CO_2_ as the evolution of our planet continues^46^. In the very distant future, a CO_2_-starved mantle would supply minimal CO_2_ to the atmosphere via mid-ocean-ridge or intraplate volcanism, possibly resulting in the eventual cooling of the surface environment.

## Methods

### Sample collection and preparation

The locations of 217 samples selected for isotopic and/or chemical analysis are shown in Supplementary Fig. 1^47–51^.

We avoid samples from the center of Syros island as they are subject to higher degrees of weathering and retrogression. Thin sections were prepared in the Yale University Department of Earth and Planetary Sciences and viewed on a Leitz Sm-Lux-Pol petrographic microscope. Samples for bulk chemical analysis were cut with a diamond rock saw to remove weathering rinds and joint surfaces, abraded using SiC paper and ultrasonicated in distilled deionized water prior to crushing. Powders for isotope analysis were extracted with a dental drill equipped with a 5-mm diamond-tipped drill bit.

### Chemical measurement and data reduction

Samples were analyzed for major elements via X-ray fluorescence on borate fused discs at SGS laboratories in Lakefield, Ontario, Canada. Minor elements were measured via X-ray fluorescence of pressed pellets. The full set of chemical measurements is available in Supplementary Data 1.

Major element oxide weight percentages were converted to moles element for input into thermodynamic modeling software. Loss-on-ignition (LOI) was corrected for ferric iron with Fe^3+^/Fe^total^ ranging from 0 to 0.15 based on modal mineralogy.

This corrected LOI was then converted to weight percent CO_2_ and H_2_O with results from point-counting of representative thin sections. Samples were point-counted in detail to constrain wt% H_2_O, and the remaining LOI was assumed to be CO_2_ (i.e., wt% CO_2_ = wt% LOI_corrected_ ‒ wt% H_2_O). For heavily altered volcanics and lightly altered oceanic crust (samples with relatively low LOI where wt% H_2_O might have a large effect), every sample was point-counted. For carbonate-bearing siliciclastics and limestones, three representative samples of each type were point-counted. These results were extrapolated to the remaining samples of that type assuming a linear relationship between wt% H_2_O and wt% LOI within each sample group such that:

2 $$\left( {{\mathrm{wt}}\% \,{\mathrm{H}}_{2}{\mathrm{O}}} \right) = s \times \left( {{\mathrm{wt}}\% \,{\mathrm{LOI}}} \right) + b,$$

where *s*_limestone_ = −0.0319 ± 0.0044, *b*_limestone_ = 1.41 ± 0.17, *s*_clastic_ = −0.1159 ± 0.1171, and *b*_clastic_ = 3.39 ± 1.85 (all errors 2σ standard deviation).

Note that the entire range of water contents is fairly small (0.01–3.8 wt% H_2_O) compared to wt% CO_2_ (1.5–43.8) (Supplementary Data 1). Taking each sample’s water content as the highest or lowest possible value from its lithologic group would not have a significant effect on any of our results or conclusions.

Rocks were sorted into four categories based on their protolith: heavily altered volcanics, lightly altered oceanic crust, carbonate-bearing siliciclastics, and limestones. Samples with SiO_2_ < 20 wt% were categorized as limestones. Carbonate-bearing siliciclastic rocks were differentiated from the meta-igneous suites based on observations of mineralogy (high abundance of micas being a particular feature of metasediments) and field context, with visible continuous compositional layering in a cohesive section indicating metasedimentary origin. Heavily altered volcanics were differentiated from lightly altered basaltic crust based on the degree of observed and back-projected carbonation, with lightly altered samples having initial CO_2_ < 10 wt%.

### Stable isotopes

Carbon and oxygen stable isotopes were analyzed in the Yale Analytical and Stable Isotope Center on either a Thermo MAT 253 KIEL IV with carbonate device or Thermo DeltaPlus XP Stable Isotope Ratio mass spectrometer with a Thermo Gasbench II interface. Consistency between the two methods was ensured via analysis of in-house standards and repeat analysis of a subset of samples. Precision is better than ± 0.1 per mille (1σ).

Carbon and oxygen isotope values are reported in δ notation relative to Vienna Pee Dee Belemnite (V-PDB) and Vienna Standard Mean Ocean Water (V-SMOW), respectively. (Fig. 2 and Supplementary Data 2). Isotope measurements represent a composite of the composition of all carbonate minerals in the sample. In most cases, this is only calcite (pseudomorphous after aragonite), but may include dolomite.

### Thermodynamic modeling

Thermodynamic calculations (including ${\mathrm{P}}{\hbox{-}}a_{{\mathrm{CO}}_{2}}$ diagrams and *P–T* pseudosections) were performed in the system Na_2_O–CaO–K_2_O–FeO–Fe_2_O_3_–MgO–MnO–Al_2_O_3_–SiO_2_–CO_2_–H_2_O–TiO_2_ using the Theriak-Domino software package^52^ with code as in ref. ^53^ and the database of ref. ^54^. Mineral and fluid activity models^55–59^ are given in Supplementary Table 1. The equations of state for H_2_O and CO_2_ are from ref. ^60^, while remaining fluid endmembers are from ref. ^61^. Additions to the default database file for full COH fluid mixing are also given in Supplementary Data 3.

### Back projection

The initial volatile content of each sample is calculated as described in the main text at low-grade *P–T* conditions of 310 °C and 0.8–1.0 GPa. These conditions are consistent with estimates of the prograde *P–T* path for the CBU^16^; critically, they are cold enough that the rock would have undergone little to no metamorphic carbon loss at this point^62^, but warm enough that the thermodynamic mixing models selected for peak *P–T* are still valid. Temperature-versus-CO_2_ loss curves calculated over a wide range of fluid compositions converge at this low *P–T* (Supplementary Fig. 2a, c), indicating that the back-projection procedure is insensitive to the fluid history of the rock.

We calculate the stable minerals in each sample at this lower *P–T* in a system with fluid in excess; we vary the composition of this fluid from extremely water-rich conditions (*X*$_{{\mathrm{CO}}_{2}}$ = 0.0001), to a pure CO_2_ fluid ($X_{{\mathrm{CO}}_{2}}$ = 1.0). The back-projected precursor composition is then taken as the composition of the solid minerals at a given set of low-grade *P*, *T*, and $X_{{\mathrm{CO}}_{2}}$ conditions. For example, a modeled precursor that contains 10 moles of aragonite, CaCO_3_, and 10 moles of dolomite, CaMg(CO_3_)_2_, would have 30 moles CO_2_. Note that the proportions of nonvolatile species (e.g., SiO_2_, CaO) are fixed to match the modern bulk composition, and only volatile components (CO_2_ and H_2_O) are variable.

In all cases, the CO_2_ content forms a flat or stepped function with broad plateaus as a function of $X_{{\mathrm{CO}}_{2}}$. In most samples, the modeled CO_2_ content is nearly constant across the entire range of conditions, insensitive to changes in *P*, *T*, or $X_{{\mathrm{CO}}_{2}}$ (Supplementary Fig. 2d). For the ten samples which do show significant variation, we conservatively select the minimum plausible initial CO_2_ content, and therefore, minimum ∆CO_2_ (Supplementary Fig. 2b). We make the reasonable assumption that samples have undergone prograde devolatilization, and therefore determine that back-projected precursor compositions with volatile contents less than the modern rock are not plausible (Supplementary Fig. 2b, d).

We note that the selected low-grade *P–T* conditions do lie near the H_2_O−CO_2_ miscibility gap^57,63^_._ However, because the back-projected rock composition is insensitive to the fluid composition ($X_{{\mathrm{CO}}_{2}}$), it is also insensitive to the location, presence, or absence of the H_2_O–CO_2_ solvus (Supplementary Fig. 2a, c).

### Calculating carbon loss

Carbon dioxide loss is calculated through a comparison of the initial and final wt% CO_2_ for each sample. We normalize to a nonvolatile chemical species (selecting SiO_2_, though any other major element would yield identical results) such that

3 $${\Delta}{\mathrm{CO}}_2 = \frac{{m_{{\mathrm{CO}}_2}^o - m_{{\mathrm{CO}}_2}^\prime }}{{m_{{\mathrm{CO}}_2}^o}} = \left[ {1 - \frac{{C_{{\mathrm{CO}}_2}^\prime }}{{C_{{\mathrm{SiO}}_2}^\prime }} \cdot \frac{{C_{{\mathrm{CO}}_2}^o}}{{C_{{\mathrm{SiO}}_2}^o}}} \right],$$

where *m*_*i*_ is the mass of a species, *C*_*i*_ is the concentration of a species, and ′ and ^*o*^ denote the final and initial rock compositions, respectively^26^. The ΔCO_2_^observed^ is calculated with *C*′_*i*_ from modern measured samples, whereas ΔCO_2_^potential^ is calculated with *C*′_*i*_ from rock compositions resulting from forward modeling as described in the main text.

### Flux calculation

We construct a model crustal stack approximately representative of the global average subducted sediments + crust. The CO_2_ loss of each lithology type is taken as the average CO_2_ loss of the corresponding type in our dataset (i.e., ΔCO_2_ = 0.032 for limestones, 0.51 for carbonate-bearing siliciclastics, 0.69 for heavily altered volcanics, and 0.46 for lightly altered basaltic crust), as is the initial weight fraction of CO_2_ for each rock type ($C_{{\mathrm{CO}}_{2}}$ = 0.39 for limestone, 0.19 for carbonate-bearing siliciclastics, 0.16 for heavily altered volcanics, and 0.06 for the altered basaltic crust.) As a weighted average using the unit thickness and densities of 2700 kg m^−3^ and 3000 kg m^−3^ for sedimentary and mafic rock types, respectively, the total CO_2_ loss fraction is 0.41 (or 41%). The total input flux into subduction zones is calculated using these thicknesses and densities in combination with global subduction length (*L* = 4.45 × 10^7^ m)^36^ and average convergence rate (*R* = 0.05 m yr^−1^)^36^ to find Eq. (4):

4 $$F_{{\mathrm{input}}} = L \cdot R{\int}_{z_1}^{z_2} {C_{{\mathrm{CO}}_2} \cdot \rho \cdot dz = 5.08\,{\mathrm{Tmol}}\,{\mathrm{CO}}_2\,{\mathrm{yr}}^{ - 1}}.$$

Multiplied by the total CO_2_ loss fraction, this gives a flux off the slab of 2.06 Tmol CO_2_ yr^−1^.

The decarbonation proportions of Central American and Tongan crustal stacks are calculated via an analogous procedure, changing only the thickness of each lithologic unit as specified in the main text. Note that we do not take into account the specific thermal structure of these end-member subduction zones, but this does not have a substantial effect on the total decarbonation predicted by our model (see main text).

### Decarbonation along a *P–T* path

The CO_2_ loss of average subducting crust is calculated along two linear prograde *P–T* paths. Both paths begin at 310 °C and 1.0 GPa; the hotter path, path A, passes through ‘peak field conditions’ of 525 °C and 1.5 GPa and proceeds up to 650 °C and 1.8 GPa. Path B passes through peak field conditions at 550 °C and 2.0 GPa, proceeding up to 650 °C and 2.4 GPa. Note that the pressures reached at 650 °C are prescribed by the assumption of a path that is linear in *P–T* space.

Beginning with the back-projected composition, each sample is forward modeled in a system with a fluid of a prescribed $X_{{\mathrm{CO}}_{2}}$. The peak equilibrium fluid compositions (ranging from $X_{{\mathrm{CO}}_{2}}$ = 0.0046–0.0081) are selected such that the rock volatile content and mineralogy predicted at peak pressure and temperature are consistent with observations of each modern sample. The CO_2_ content of each rock is determined at steps of 1 °C along the prograde *P–T* paths, passing through the peak field conditions, and continuing up to 650 °C.

Recall that we model 12 representative samples (three from each lithologic group). In nine samples, the fluid composition is fixed at a given $X_{{\mathrm{CO}}_{2}}$ with fluid in excess. The three samples of lightly altered oceanic crust are treated slightly differently. In the case of these rocks, back projection at $X_{{\mathrm{CO}}_{2}}$ = ~0.0001 gives plausible initial compositions and, as described above, we select these lowest CO_2_ compositions to be conservative. However, the observed mineralogy and final volatile content of these rocks require $X_{{\mathrm{CO}}_{2}}$ closer to ~0.01, thus the fluid composition is allowed to evolve from $X_{{\mathrm{CO}}_{2}}$ = ~0.0001 to $X_{{\mathrm{CO}}_{2}}$ = ~0.01 as *P* and *T* increase from starting to peak conditions. The other nine samples can achieve the most conservative back-projected CO_2_ content and the observed modern mineralogy and CO_2_ content at the same $X_{{\mathrm{CO}}_{2}}$, so they are modeled with a fixed fluid composition.

Note that each of the 12 samples requires a slightly different fluid composition for the differing *P–T* paths (A and B) in order to match our observations. That is, the system is underdetermined: given our observations of mineralogy and volatile content and two of the set {*P, T*, $X_{{\mathrm{CO}}_{2}}$}, we can constrain the third parameter. For example, for sample JAGSY-147Y, the fluid composition which matches the observed assemblage would be $X_{{\mathrm{CO}}_{2}}$ = 0.0073 at 525 °C and 1.5 GPa, whereas at conditions of 550 °C and 2.0 GPa the equilibrium fluid would need to be $X_{{\mathrm{CO}}_{2}}$ = 0.0056 to produce the same assemblage.

The CO_2_ content of each selected rock is calculated at steps of 1 °C along both *P–T* paths. The incremental CO_2_ released at each *P–T* step is calculated by difference with the preceding steps. The average mass change of CO_2_ of each lithologic type is combined with the same unit thicknesses and densities from the global flux calculation to arrive at a weighted average degree of decarbonation for the stack.

The three samples representing each lithology were forced to match the average decarbonation of their whole lithologic group by multiplying by some constant value. These are small corrections, the largest being an adjustment of the CO_2_ loss fraction of the three representative metamorphosed limestones, multiplying by ~0.7 to correct from 4.5% CO_2_ loss to the total group average of 3.2%. In this way, the total decarbonation of the crustal section is forced to equal 41% at peak *P–T* conditions. The same factor is used in the forward modeling to higher *P–T* conditions: for example, the three metamorphosed limestones average 6.5% decarbonation at 650 °C, which is multiplied by 0.7 to arrive at 4.5% decarbonation for the whole suite of metamorphosed limestones. The correction factors for the three remaining lithologic types are all between 0.95 and 1.05 for both *P–T* paths. Similarly, the initial average wt% CO_2_ of each lithologic group is set to match the entire dataset. Supplementary Fig. 4 shows the mass change of the twelve representative samples along each *P–T* path and the adjusted average mass change for each lithologic group.

## Supplementary information

Supplementary Information

Description of Additional Supplementary Files

Supplementary Data 1

Supplementary Data 2

Supplementary Data 3

## Data Availability

The authors declare that the data supporting the findings of this study are available within the paper and its supplementary information files.
