# Supplementary material for: Pervasive subduction zone devolatilization recycles CO2 into the forearc
Source: Nat Commun. 2020 Dec 4;11:6220. doi: 10.1038/s41467-020-19993-2 (PMC7718257; doi:10.1038/s41467-020-19993-2)
Supplement: Supplementary file 1 — Supplementary Information [file 41467_2020_19993_MOESM1_ESM.pdf]

Supplementary Information

Pervasive subduction zone devolatilization recycles CO_2 into the forearc

E.M. Stewart* and Jay J. Ague

*corresponding author, email: estewart@caltech.edu

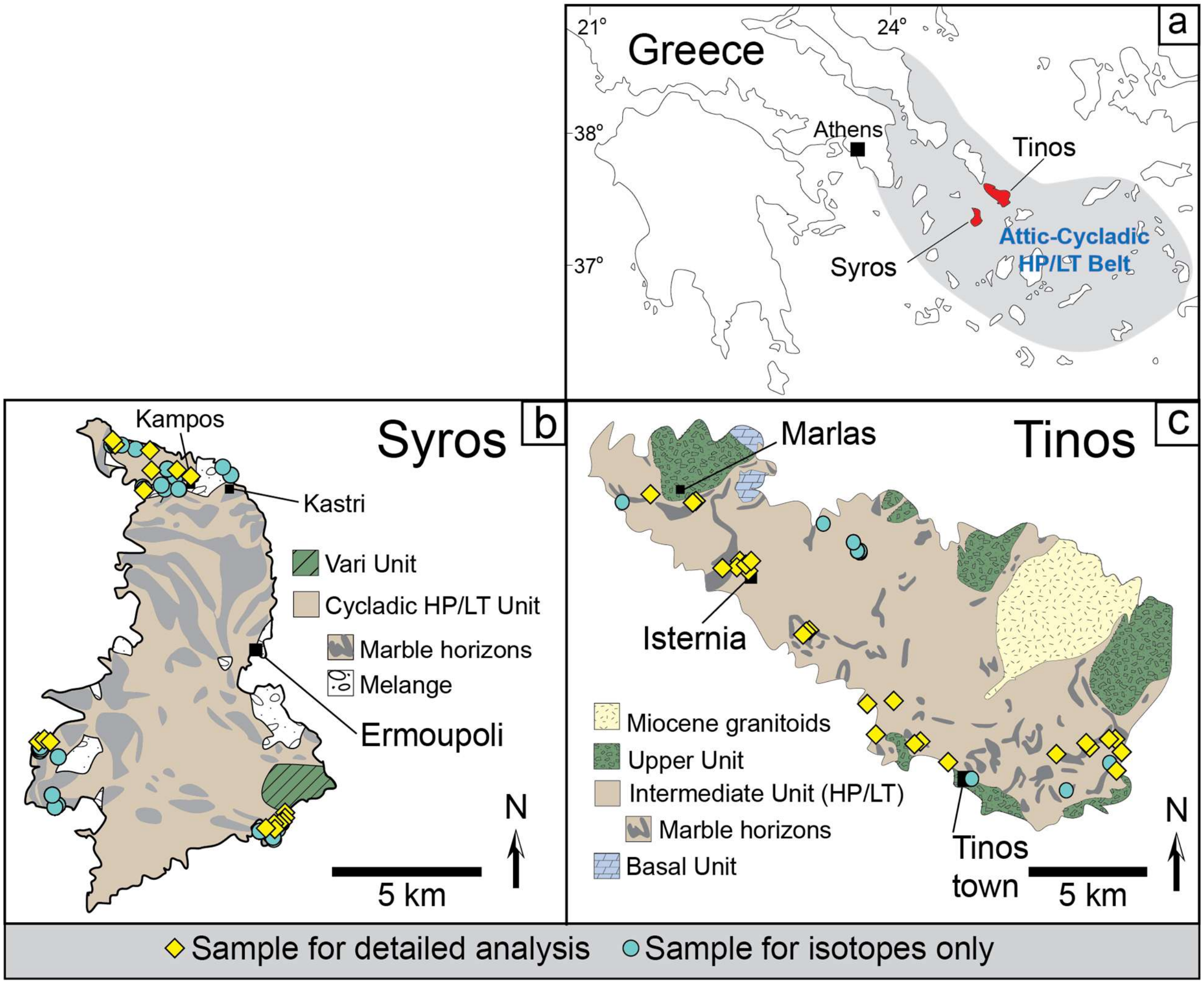

Supplementary Fig. 1: Sample locations The 217 samples selected for detailed chemical analysis and/or isotope work are shown. **a,** regional map showing location of the CBU **b**, geologic map of Syros. **c,** geologic map of Tinos. All panels are modified from ref.[26] and refs.[47-51] in the main text.

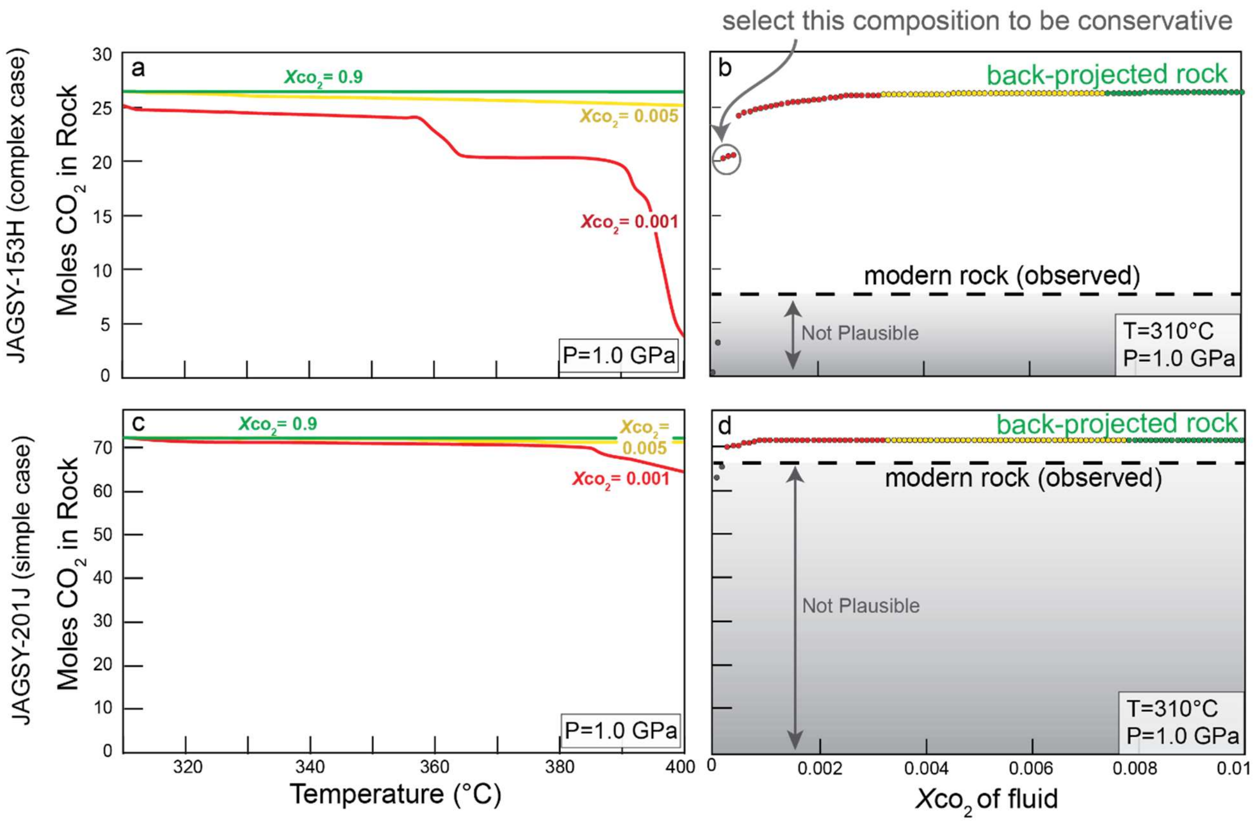

Supplementary Fig. 2 Back-projection method. The carbon content of a rock is shown as a function of temperature in systems with fluid compositions from X_{CO_2} = 0.001 to X_{CO_2} = 0.9 for samples JAGSY-153H (**a**) and JAGSY-201J (**c**). At low temperature, the molar CO_2 content of all fluid paths converges to a similar value, indicating the back-projected composition is relatively insensitive to X_{CO_2}. In some samples (e.g. JAGSY-153H) there are multiple valid back-projected values (**b**), and we select the minimum molar CO_2 to be conservative in carbon loss calculations. Most samples (e.g., JAGSY-201J) have only one broad plateau of plausible back-projected values (**d**). Initial CO_2 concentrations less than the modern observed CO_2 content are considered implausible.

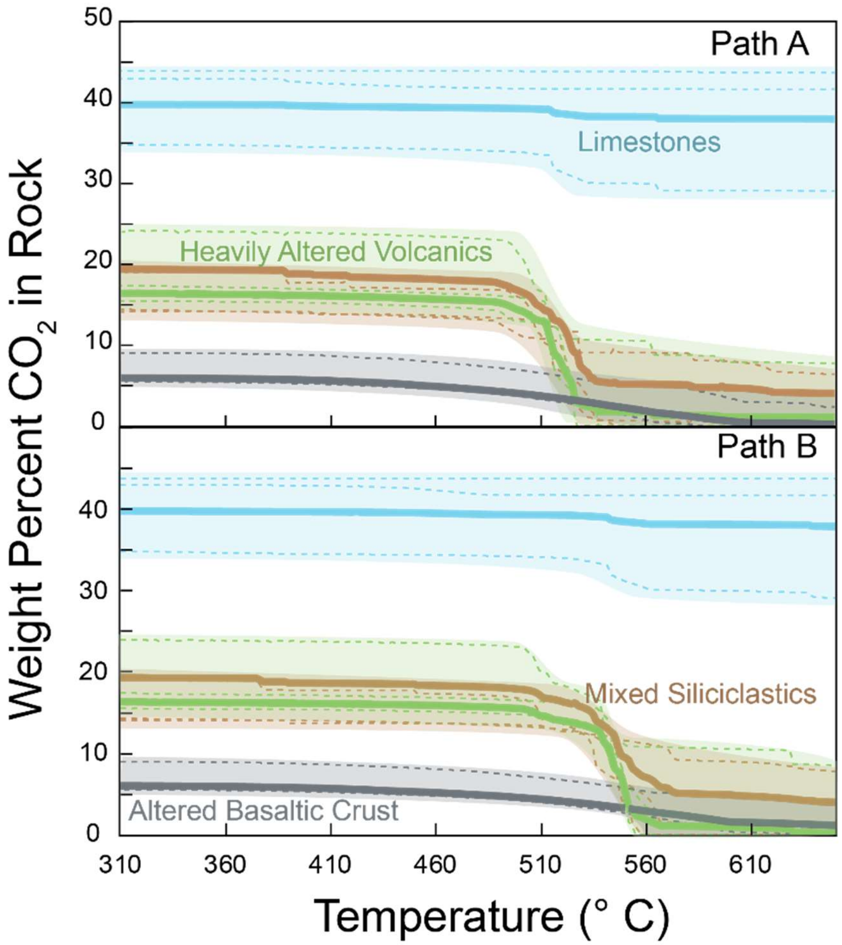

Supplementary Fig. 3: CO_2 loss along a *P-T* Path Curves show the weight percent CO_2 in three representative samples of each lithologic type (dashed lines) and the corrected average for that type (solid lines). The CO_2 pulse around 500 to 550 ºC is dominated by the dolomite-out reaction in heavily altered volcanics and mixed siliciclastic rocks, occurring at slightly different conditions along P-T paths A and B.

Supplementary Table 1: Mineral and fluid mixing activity models

Source	Phase(s)
Green *et al.* (2016)	amphibole, clinopyroxene
Holland & Powell (1998)	dolomite-ankerite, magnesite-siderite
Holland & Powell (2003)	COH fluid, plagioclase
Holland & Powell (2011)	epidote
White *et al.* (2000)	ilmenite
White *et al.* (2014)	garnet, biotite, chlorite, chloritoid, white mica
ideal mixing	carpholite, prehnite, pumpelleyite, sudoite, talc
