# Supplementary material for: Pervasive subduction zone devolatilization recycles CO2 into the forearc
Source: Nat Commun. 2020 Dec 4;11:6220. doi: 10.1038/s41467-020-19993-2 (PMC7718257; doi:10.1038/s41467-020-19993-2)
Supplement: Supplementary file 2 — Description of Additional Supplementary Files [file 41467_2020_19993_MOESM2_ESM.pdf]

Description of Additional Supplementary Files

File Name: Supplementary Data 1
Description: A Supplementary Data table containing measured bulk chemistry, back-projected volatile contents, observed mineralogies, and calculated values (e.g., Delta CO_2, and equilibrium activity of CO_2) for 56 samples studied in detail.

File Name: Supplementary Data 2
Description: Supplementary Data table including 218 carbon and oxygen isotope analyses and corresponding latitude/longitude of each sample.

File Name: Supplementary Data 3
Description: A text file containing additions to the default thermodynamic database for Theriak-Domino. These additions allow calculations with full COH fluid mixing.
